# Clopidogrel-induced drug-induced hypersensitivity syndrome following percutaneous coronary intervention: a case report of therapeutic dilemma and management strategy

**DOI:** 10.3389/fphar.2025.1695177

**Published:** 2026-02-10

**Authors:** Sikun Wang, Jiang Zhou, Yuanhua Sun

**Affiliations:** 1 Department of Gastroenterology, Taihe Hospital, Hubei University of Medicine, Shiyan, Hubei, China; 2 Department of Emergency Medicine, Taihe Hospital, Hubei University of Medicine, Shiyan, Hubei, China; 3 Department of Spinal Surgery, Taihe Hospital, Hubei University of Medicine, Shiyan, Hubei, China

**Keywords:** clopidogrel, drug-induced hypersensitivity syndrome, DRESS syndrome, percutaneous coronary intervention, dual antiplatelet therapy, stent thrombosis, indobufen

## Abstract

**Background:**

Drug-induced hypersensitivity syndrome (DIHS), also known as drug reaction with eosinophilia and systemic symptoms (DRESS), is a rare but potentially life-threatening adverse drug reaction. Clopidogrel induced DHS following percutaneous coronary intervention (PCI) presents a unique therapeutic challenge due to the critical need for dual antiplatelet therapy to prevent stent thrombosis.

**Case summary:**

A 70-year-old male underwent emergency PCI with multiple stent implantation for unstable angina with three-vessel coronary disease. Standard dual antiplatelet therapy (aspirin 100 mg daily and clopidogrel 75 mg daily) was initiated post-procedure. One hour after PCI, the patient developed anaphylactic shock initially attributed to contrast agent allergy. Two weeks post-discharge, he presented with recurrent skin rash, hypotension, gastrointestinal bleeding, fever, and eosinophilia, consistent with DIHS. Multidisciplinary consultation confirmed clopidogrel-induced DIHS using established diagnostic criteria. Despite the high risk of stent thrombosis, dual antiplatelet therapy was discontinued due to life-threatening complications. The patient was successfully managed with indobufen and low dose corticosteroids, resulting in complete symptom resolution.

**Conclusion:**

This case highlights the diagnostic complexity of clopidogrel-induced DIHS and presents the detailed account of successful management of it. Early recognition using standardized diagnostic criteria and multidisciplinary management are crucial for patient outcomes.

## Introduction

Drug-induced hypersensitivity syndrome (DIHS), also referred to as drug reaction with eosinophilia and systemic symptoms (DRESS) syndrome, represents a severe, potentially fatal adverse drug reaction characterized by the classic triad of fever, skin eruption, and internal organ involvement (Cacoub et al., 2011; Cho et al., 2017). The syndrome typically manifests 2–6 weeks after drug initiation, with a reported mortality rate of 5%–10% (Hama et al., 2022; Wei et al., 2024). Pathophysiology involves complex T-cell-mediated delayed hypersensitivity reactions, which are usually triggered by the formation of new antigen complexes between reactive drug metabolites and organism protein. Activated T lymphocytes produce large amounts of pro-inflammatory cytokines, causes systemic inflammation and organ failure associated with DRESS (Pichler, 2019; Miyagawa and Asada, 2021; Calle et al., 2023). Clopidogrel, a thienopyridine P2Y12 receptor antagonist, is an essential component of dual antiplatelet therapy (DAPT) following PCI. It is usually well tolerated and rarely causes severe allergic reactions, including DIHS. The active metabolites of clopidogrel, especially two-ox-clopidogrel, trigger T-cell immune responses through interactions with cellular proteins (Dansette et al., 2013; Kazui et al., 2010).

The clinical challenge in post-PCI settings is profound: discontinuation of DAPT within the first 3 months significantly increases the risk of stent thrombosis, a potentially fatal complication occurring in 0.5%–2% of patients (Iakovou et al., 2005; Levine et al., 2016). However, the continued use of pathogenic drugs in established DIHS can lead to progressive multiple organ failure and death. This case report aims to conduct a comprehensive analysis of clopidogrel-induced DIHS using the established diagnostic framework and propose a personalized treatment approach that balances the risks of thrombosis and allergy.

## Case summary

A 70-year-old male with a medical history of hypertension (controlled with enalapril 20 mg daily) and type 2 diabetes mellitus (managed with metformin 1,000 mg daily) presented to the emergency department with unstable angina. His HbA1c was 6.8%, and blood pressure was well-controlled at 135/80 mmHg. The patient had no known drug allergies or previous adverse drug reactions. Emergent cardiac catheterization revealed severe three-vessel coronary artery disease with critical stenoses: 95% stenosis of the left anterior descending artery (LAD), 90% stenosis of the left circumflex artery (LCX), and 85% stenosis of the right coronary artery (RCA), the SYNTAX I score tends to reach the boundary between low and medium-risk stratification. The patient was immediately given loading doses including 300 mg of aspirin and 600 mg of clopidogrel. Subsequently, he successfully underwent PCI with implantation of three second-generation drug-eluting stents (Firebird2 3.5*2.3 mm) with final TIMI three flow and no periprocedural complications. Standard post-PCI medications were initiated: Aspirin 100 mg daily, Clopidogrel 75 mg daily, Rosuvastatin 20 mg daily, Metoprolol 50 mg twice daily, Enalapril 20 mg daily (continued). Approximately 1 hour post-procedure, the patient developed acute onset generalized urticaria, severe hypotension (blood pressure 75/45 mmHg), and bronchospasm consistent with anaphylactic shock. The reaction was initially attributed to iodinated contrast agent hypersensitivity given the temporal relationship. Emergency treatment included intravenous epinephrine 0.5 mg, methylprednisolone 125 mg IV, diphenhydramine 50 mg IV and aggressive fluid resuscitation. The patient responded rapidly to treatment with complete resolution of symptoms within 2 h. He was monitored in the cardiac care unit for 48 h and discharged home on standard post-PCI medications.

**FIGURE 1:**
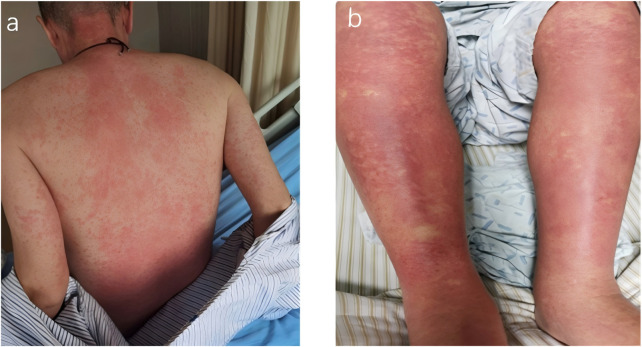
**(a,b)** The patient presented with diffuse, concomitant, non-pruritic erythema, involving the trunk and limbs, without extensive epidermal necrosis and peeling or multiple mucosal erosions.

**FIGURE 2:**
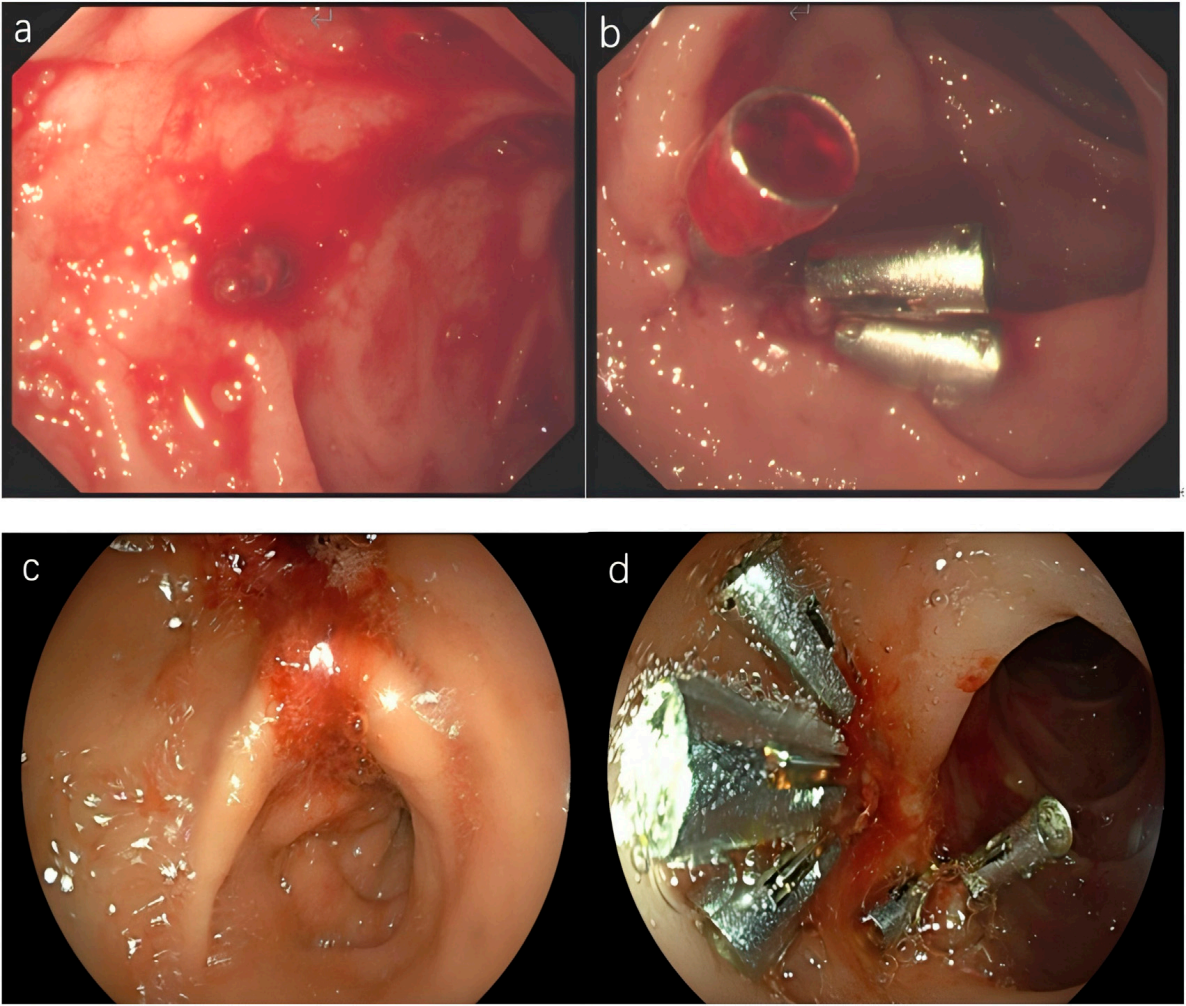
**(a,b)** The first gastroscopy of the patient revealed a thrombus head in the upper descending part of the duodenum, accompanied by active bleeding. Three titanium clips were used for clamping and hemostasis. **(c,d)** The patient’s second gastroscopy revealed a local thrombus head in the upper descending part of the duodenum, accompanied by active bleeding. Five titanium clips were used again for clamping and hemostasis.

The patient presented to the emergency department with progressive fusion rashes involving the trunk and extremities on the day 14 post-PCI (Figure 1). The rash was nonpruritic and associated with low-grade fever (temperature 38.2 °C), and development of additional symptoms on the day 16 post-PCI including persistent fever (up to 39.1 °C), hypotension episodes (systolic BP 85–95 mmHg), persistent hematemesis and melena, facial edema, generalized malaise and confusion, Comprehensive laboratory investigations were performed (Table 1), Chest X-ray confirmed bilateral lower lobe infiltrates, abdominal CT confirmed mild hepatomegaly, and echocardiography confirmed normal left ventricular function, no pericardial effusion. Gastroscopy revealed gastric and duodenal ulcers accompanied by active bleeding. We attributed the bleeding mainly to aspirin-related gastrointestinal injury and early DAPT amplified it. Subsequent endoscopic treatment successfully stopped the bleeding. (Figure 2). The DIHS/DRESS diagnostic criteria usually adopted RegiSCAR criteria and our patient achieved a definite DRESS diagnosis (score 9), The probable causality score. Supports clopidogrel as the likely pathogenic factors. So we organized a multidisciplinary team including cardiology, hematology, dermatology, gastroenterology, and clinical pharmacology was assembled. The team faced a critical therapeutic dilemma including risk of continuing clopidogrel and discontinuing clopidogrel.

**TABLE 1:** Laboratory findings during DIHS episode.

Parameter	Day 16	Day 18	Day 21	References range
Hematology				
Hemoglobin (g/dL)	8.2	7.8	9.1	12.0–16.0
Platelet count (×10^3^/μL)	156	142	178	150–450
White blood cells (×10^3^/μL)	12.8	15.2	10.9	4.0–11.0
Eosinophils (×10^3^/μL)	2.1	2.8	1.9	<0.5
Eosinophil percentage (%)	16.4	18.4	17.4	<4.0
Hepatic function				
ALT (U/L)	156	198	142	10–50
AST (U/L)	142	174	128	10–40
Total bilirubin (mg/dL)	2.1	2.8	1.9	0.3–1.2
Alkaline phosphatase (U/L)	145	167	139	44–147
Renal function				
Creatinine (mg/dL)	1.2	1.4	1.1	0.8–1.3
BUN (mg/dL)	28	32	24	7–20
Inflammatory markers				
CRP (mg/L)	45.6	52.1	38.9	<3.0
ESR (mm/h)	68	75	59	<30
hs-cTnT (μg/L)	0.015	0.007	0.011	<0.02

ALT, alanine aminotransferase; AST, aspartate aminotransferase; BUN, blood urea nitrogen; CRP, C-reactive protein; ESR, erythrocyte sedimentation rate; hs-cTnT, cardiac troponin T.

On the 18th day after PCI, due to the dual risks of life-threatening DIHS/DRESS and persistent upper gastrointestinal bleeding. We believe that in the process of extensive corticosteroid treatment, priority should be given to controlling allergic reactions and preventing rebleeding. After reaching a multidisciplinary consensus, clopidogrel and aspirin was discontinued. As an individualized, risk-mitigating alternative, indobufen 100 mg twice daily was initiated, prednisolone 1 mg/kg/day (70 mg daily) for DIHS management, proton pump inhibitors protect the gastrointestinal tract (omeprazole 40 mg twice daily) and supportive care including fluid management and monitoring. The patient rapidly improvement in skin rash within 48 h, the fever relieved on the 21st day, the eosinophil count normalized on the 23rd day, gastrointestinal bleeding stopped, and liver function improved, Troponin T monitoring has always been below 0.02 μg/L. During the subsequent 24-month long-term follow-up, the patient continued indobufen treatment and tolerated it well. DIHS achieved complete remission without sequelae, coronary angiography indicated that the stent was unobstructed without stenosis and other cardiovascular events occurred, no gastrointestinal bleeding occurred, eosinophil counts, liver and renal function normalized and physical functional status returned to the baseline level (Table 2).

**TABLE 2:** Long-term follow-up data.

Parameter	1 Month	3 Months	6 Months	12 Months	24 Months
Clinical status					
NYHA Class	II	I	I	I	I
Skin lesions	Resolved	None	None	None	None
GI symptoms	Mild	None	None	None	None
Laboratory					
Eosinophils (×10^3^/μL)	0.45	0.28	0.32	0.31	0.29
ALT (U/L)	45	32	28	31	29
Creatinine (mg/dL)	1	0.9	1	1.1	1
Cardiac assessment					
Ejection fraction (%)	55	58	60	58	60
Stress test	-	Normal	-	Normal	-
Angiography	-	-	No thrombosis	-	No thrombosis
hs-cTnT (μg/L)	0.017	0.005	0.008	0.011	0.003

## Discussion

Clopidogrel-induced DIHS is a complex immune phenomenon involving multiple pathways. This drug is widely metabolized in the liver through cytochrome P450 enzymes, especially CYP2C19, CYP3A4 and CYP1A2 enzymes involved in metabolite production (Kazui et al., 2010; Zhang et al., 2012). Among them, the CYP2C19 gene polymorphism may affect the risk of DIHS occurrence. Patients with CYP2C19*2 and *3 alleles (poor metabolism) may have altered metabolite profiles, which may regulate immunogenicity and clinical phenotypes (Mega et al., 2009). Although genotyping was not performed in this case, we acknowledge its prospective value for risk stratification and post-event evaluation in clopidogrel hypersensitivity, and we encourage future studies to integrate pharmacogenomics into prediction and prevention frameworks for DIHS/DRESS. The diagnosis of clopidogrel-induced DIHS presents several challenges including delayed Onset, initial misattribution, DIHS. Symptoms can overlap with other postoperative complications, including infection, contrast-induced nephropathy or surgery-related bleeding. So the application of standardized diagnostic criteria is crucial. The DIHS/DRESS diagnostic criteria usually adopted RegiSCAR criteria (Table 3) and our patient achieved a definite DRESS diagnosis (score 9), This scoring system has 94% specificity and 85% sensitivity for DRESS diagnosis (Kardaun et al., 2013; Sasidharanpillai et al., 2022). The Naranjo scores of the three suspected drugs were all within the “possible” range (Naranjo et al., 1981) (Table 4). Therefore, recognizing the limitations of Naranjo in terms of delayed potentially fatal reactions, we added the ADDRESS (Table 5) algorithm to further compare the three suspected drugs (clopidogrel, aspirin, and iodine contrast agent) (Stewart and Ramírez, 2024). Clopidogrel aligned most closely with a 2–6-week latency, eosinophilia, multi-organ involvement typical of DIHS/DRESS, and improvement after withdrawal. Aspirin remains a possible factor, but its consistency with the systemic manifestation dominated by eosinophils and the temporal relationship is relatively weak. The contrast agent well explains the immediate allergic reaction after PCI, rather than the delayed DIHS/DRESS occurrence trajectory. While residual uncertainty remains, the aggregate clinical and temporal evidence favored clopidogrel as the primary trigger.

**TABLE 3:** DIHS/DRESS diagnostic criteria (RegiSCAR diagnostic).

Criteria	Score	Patient status
Fever ≥38.5 °C	0 (−1 if absent, 0 if 38.0 °C–38.4 °C, +1 if ≥ 38.5 °C)	+1 (39.1 °C)
Enlarged lymph nodes	0 (+1 if present, 0 if absent)	0 (absent)
Eosinophilia	+2 (+1 if 700–1,499/μL, +2 if ≥ 1,500/μL)	+2 (2,100/μL)
Atypical lymphocytes	0 (+1 if present, 0 if absent)	0 (absent)
Skin involvement	+2 (+1 if rash extent >50%, +2 if>50% with edema/infiltration)	+2 (>50% with edema)
Organ involvement	+2 (+1 for one organ, +2 for ≥2 organs)	+2 (liver, kidney, lung)
Resolution ≥15 days	0 (−1 if < 15 days, 0 if unknown, +1 if ≥ 15 days	+1 (21 days)

Total RegiSCAR, Score: nine points = Definite DRESS (Score interpretation: <2 = no case, 2–3 = possible, 4–5 = probable, >5 = definite).

**TABLE 4:** Naranjo causality assessment.

Question	Clopidogrel	Aspirin	Iodinated contrast
Previous conclusive reports on this reaction?	+1	+1	0
Did the adverse event appear after suspected drug?	+2	+2	+2
Did reaction improve when drug discontinued?	+1	0	0
Did reaction reappear when drug readministered?	0	0	0
Alternative causes for the reaction?	−1	−1	−1
Did reaction occur after placebo?	0	0	0
Drug detected in blood/body fluids?	0	0	0
Reaction more severe with increased dose?	0	0	0
Previous similar reaction to same/similar drugs?	0	0	0
Adverse event confirmed by objective evidence?	+1	+1	+1
Total score	4	3	2
Naranjo Category	Possible	Possible	Possible

≥9 definite, 5–8 probable, 1–4 possible, ≤0 doubtful.

**TABLE 5:** ADDRESS-informed multi-suspect causality scoring.

Component	Clopidogrel	Aspirin	Iodinated contrast
Lantency	+2	+1	−1
Pharmacokinetics	+1	0	−1
Pre-/Re-challenge	0	0	0
De-challenge	+1	0	0
Drug risk level for DRESS	+1	0	−1
Alternative causes	+1	−1	−1
Total	6	0	−4

Scoring rubric used in this manuscript: Latency +2/+1/0/−1; Pharmacokinetics +1/0/−1; Re-challenge +2/0; De-challenge +1/0/−1; Drug-risk +1/0/−1; Alternatives +1/0/−1. Higher totals indicate higher likelihood as the culprit drug.

We also conducted a comprehensive literature review of previously reported cases of clopidogrel-induced DIHS/DRESS. The consensus across reports indicates that immediate discontinuation of the offending agent is the primary therapeutic step, followed by systemic corticosteroids and supportive care for severe systemic involvement. To maintain essential antiplatelet protection, alternative agents with distinct mechanisms should be selected and closely monitored. In China, prasugrel is not yet available. Previous case reports have described immune cross-reactivity between clopidogrel and prasugrel after drug substitution (Siu et al., 2016). Ticagrelor, a reversible and chemically distinct P2Y12 inhibitor, is a guideline-recommended first-line alternative. Several reports have documented successful use of ticagrelor in patients with clopidogrel hypersensitivity without recurrent allergic reactions (Manchette and Drucker, 2014; Harris and Coons, 2014). However, isolated cases of cross-reactive hypersensitivity after switching to ticagrelor have been reported (Chin et al., 2015), possibly due to shared P2Y12 receptor inhibition during an immunologically active period. Although ticagrelor and clopidogrel have different chemical structure and a lower theoretical risk of cross-reaction, there is uncertainty of reactivation of allergic reactions when re-exposed to the P2Y12 pathway during the highly active immune period. In parallel, the patient has endoscopically confirmed upper gastrointestinal bleeding, the combination of potent P2Y12 inhibitors would significantly amplify the risk of bleeding in the situation of systemic hormone therapy. Clinical trials from china have demonstrated that indobufen can safely replace aspirin as part of DAPT after DES implantation (Shi et al., 2022), reducing bleeding risk without compromising ischemic protection. Aspirin monotherapy or dose escalation was inappropriate due to heightened gastrointestinal bleeding risk. Facing the dual threat of life-threatening DIHS/DRESS progression and active gastrointestinal bleeding, the multidisciplinary team determined that these complications outweighed the short-term risk of stent thrombosis and serial troponin measurements remained within the normal range after PCI, indicating no peri-procedural myocardial injury or acute stent thrombosis. This biochemical stability provided supportive evidence for the temporary discontinuation of DAPT during management of life-threatening DIHS/DRESS. Considering the regional availability, gastrointestinal safety, and distinct pharmacologic mechanism of indobufen (COX inhibition), after obtaining the informed consent of the patients for the treatment plan and conducting a common risk assessment, we ultimately adopted indobufen monotherapy as a transitional individualized strategy. It should be emphasized that although indobufen has been used in China as a substitute for aspirin in DAPT following PCI, randomized data supporting its immediate monotherapy use in ACS patients are limited. We therefore do not recommend indobufen monotherapy as a universal replacement for guideline-directed DAPT, but as a feasible contingency when life-threatening DIHS/DRESS and active bleeding coexist. If the ischemic risk increases during follow-up, a ticagrelor-based regimen (including short-term DAPT followed by monotherapy) remains our designated alternative strategy.

The patient felt uncomfortable due to the appearance of DRESS and persistent active bleeding, and was also worried that if the treatment plan was adjusted, it might cause thrombosis within the stent. After a comprehensive understanding of the multidisciplinary consultation opinions and individualized treatment plans, the patient agreed to adopt the indobufen treatment plan, which focuses on controlling allergic reactions and preventing bleeding. During the long-term follow-up period, the symptoms were effectively relieved and no ischemic events occurred. The patient expressed satisfaction with the treatment effect.

## Conclusion

This case report presents the detailed account of successful management of clopidogrel-induced DIHS. It highlights multiple clinical values: achieving objective diagnosis and causal relationship assessment through standardization tools, and the importance of multidisciplinary collaboration in the management of complications after complex PCI. During the treatment process, it is necessary to emphasize the necessity of early identification, timely drug withdrawal, reasonable supportive treatment and individualized alternative antiplatelet regimens selection. The feasibility and safety of the alternative strategies are verified through systematic risk-benefit analysis and long-term follow-up. Clinicians should remain highly vigilant about delayed drug hypersensitivity in patients after PCI and promptly activate alternative treatment options when standard treatment contraindications occur. Future research should focus on pharmacogenomics, develop predictive models for DIHS risk, conduct comparative effectiveness studies on alternative antiplatelet strategies, and establish a complete set of practical guidelines and expert consensus for managing this rare but serious complication.

## Data Availability

Because the dataset concerns a single patient and includes potentially identifiable clinical information, the raw data are not publicly available to protect privacy and comply with institutional/ethical requirements. Upon reasonable request, and subject to a Data Use Agreement and IRB approval/waiver consistent with the consent obtained, a de-identified minimal dataset (case timeline, aggregated key laboratory values, causality scores “RegiSCAR/Naranjo/RUCAM” and medication summary; redacted key imaging frames if applicable) may be shared. Data are provided for research use only with no onward sharing or re-identification. Requests should be directed to the corresponding author.
